# Investigation of the Process Optimization for L-PBF Hastelloy X Alloy on Microstructure and Mechanical Properties

**DOI:** 10.3390/ma18081890

**Published:** 2025-04-21

**Authors:** Phuangphaga Daram, Masahiro Kusano, Makoto Watanabe

**Affiliations:** Research Center for Structural Materials, National Institute for Materials Science, Tsukuba 305-0047, Japan; daram.phuangphaga@nims.go.jp (P.D.); kusano.masahiro@nims.go.jp (M.K.)

**Keywords:** laser powder bed fusion process, Hastelloy X alloy, microstructure, mechanical properties, FEM analysis

## Abstract

The purpose of this study is to investigate the effects of process parameters on the microstructure and mechanical properties of the Hastelloy X (HX) alloy using a laser powder bed fusion (L-PBF) process. A combined experimental and numerical approach was used to evaluate the influence of the energy density distribution and temperature evolution on the microstructure, defects, and mechanical properties. After the specimens were built on SUS304 substrate by the L-PBF, the microstructure and defects in the specimens were analyzed by SEM and EBSD analysis methods, and then the hardness and the tensile tests were performed. The cooling rate under different laser conditions was obtained by the finite element method (FEM). The results show that a low volume energy density (VED) was applied to the unmelted powder particles, and a high energy density resulted in spherical defects. In addition, the microstructures were found to coarsen with increasing the energy density along with a tendency to strengthen the (001) texture orientation in both x–y and x–z planes. Compared to the parts with the thermal history from numerical results, the low cooling rate with high energy density had larger crystal grains elongated along the building direction, coarser sub-grains, resulting in a reduction in microhardness and yield strength together with an increase in elongation for the L-PBF HX alloy. The presented results provide new insights into the effects of parameters and the cooling rates. It can play an important role in optimizing the L-PBF processing parameters, identifying the cause of defects, and controlling the cooling rates for the crystallographic texture in such a way as to guide the development of better metrics for designing processing parameters with the desired mechanical properties.

## 1. Introduction

Hastelloy X (Ni-Cr-Fe-Mo), also known as solid solution strengthened nickel-base alloys, offers a combination of mechanical strength and oxidation resistance, which makes it suitable for applications at elevated temperatures and in aggressive chemical environments. It is commonly used in gas turbine engines, industrial furnace components, and other high-temperature applications where oxidation resistance and mechanical strength are critical [1,2,3,4]. Traditional processes are unable to manufacture the requirement of complex structures due to long production cycles and increased costs. Additive manufacturing, also known as 3D printing, has been increasingly explored for the production of complex and high-performance materials.

Laser powder bed fusion (LPBF) is a popular additive manufacturing process used to create layer-by-layer with a high thermal gradient and rapid cooling rate (~106 K/s) up to ambient temperature [5]. The quality of the formed parts is determined by the heating/cooling rate, the thermal history, and the shape of the melt pool caused by the energy density of the energy input such as the specific process parameters of laser power, scan speed, layer thickness, and hatch spacing or beam diameter. In recent years, significant progress has been made with the LPBF-produced HX alloys in terms of the process scanning speed, hatch spacing, laser energy density, build orientation, and scanning strategy, which affect the microstructure, density, defects, and properties. The optimized process parameters are the most important to study, and there may be multiple factors for the rapid solidification of non-equilibrium during the L-PBF process. Therefore, it is necessary to investigate the effect of process parameters on the mechanical properties and microstructures of the HX specimens. The high laser power and low scanning speed that produced microcracks in LPBF HX alloys [1,6], together with insufficient laser energy input, could be attributed to the high scanning speed and low laser power, leading to the formation of the lack of fusion (LOF) defects and poor mechanical properties [7,8]. Kitano et al. developed the quick and inexpensive process parameter optimization framework to explain the defect-free conditions using single-track test and thermal elastoplastic analysis. The results showed that keyholing occurred under high laser power and low scan speed, balling occurred under high laser power and high scanning speed, and residual un-melted areas occurred under low laser power and high scanning speed [7]. Moreover, Esmaeilizadeh et al. found that extremely high (>1300 mm/s) and low (<550 mm/s) scanning speeds resulted in the lack of fusion and keyhole defects, respectively, which were found to reduce the tensile strength and ductility of the L-PBF HX specimens [2,9].

The unique microstructure of the LPBF structures is a factor influencing the physical properties, which are different from those of the cast material. The L-PBF microstructure exhibits supersaturated solid solution with micro-segregation along the dendrite arms as a result of the high cooling rate. The L-PBF process parameters define the texture intensity and grain aspect ratio [9,10]. The relationship between process and microstructure for L-PBF HX alloy is one of the most interesting and challenging in this field due to the complexity of the process and many factors on the parameters process. The lack of understanding of the melting and solidification process under rapid cooling creates a major obstacle to the adoption of the L-PBF in HX manufacturing. Hibino et al. presented that the mechanical properties of the samples varied depending on their texture on L-PBF HX alloy [3]. The effect of laser power on the microstructure formation of Hastelloy X by Monte-ro-Sistiaga et al. presented a decreased hardness and yield strength due to larger cell and grain formation with a more pronounced texture when applying high power laser with a top-hat [11]. Similarly to Huang et al., they showed that the HX alloy exhibited strong anisotropy in yield strength, with higher strength along the transverse direction than the building direction. The morphologically anisotropic columnar grains with finer boundary spacing under transverse stress are shown to be the main reason [4]. The complexity of these phenomena makes it difficult to select the proper combinations of processing parameters, to achieve the desired microstructure, particularly grain size, to design materials with demanding performance. The thermal history of the printed part as it is built layer-by-layer has an effect on defects (solidification cracking) and microstructure evolution [7,8,12,13,14]. The process parameters influence the thermal gradients and the cooling rate during the process, which can lead to significant changes in the properties of a qualified part. More specifically, the measurements of the thermal gradient and the solid–liquid interface velocity are required.

A finite element model has been suggested to calculate the cooling rate, temperature gradient, and solidification rate in the melt pool [8,9]. The solidification cracking of L-PBF HX specimens in the single-track formation tests for different laser conditions, and the degree of change in the plastic strain during the solidification process obtained using the thermal elastoplastic analysis model were investigated in Kitano et al. [8]. Most of the experimental research has focused on the relationship between processing parameters and consequential defects in laser additive manufacturing of nickel superalloys [1,7,8,9,10,11]. However, there are few studies to optimize the process parameters of the L-PBF HX alloy which it is still interesting to study the solidified grain structure, especially the grain size, and to obtain the desired microstructure and properties by selecting the processing parameters. Therefore, numerical analysis combined with experimental results have been selected as a feasible method to obtain L-PBF HX structure by a careful selection of the processing parameters which are closely related to thermal gradient (G), cooling rate (GxR), and solidification rate (R).

The aim of the present study is to exploit the L-PBF fabrication using HX powder under different processing parameters including scanning speed, and laser power, which were carried out to investigate the effect of processing parameters on defects, microstructure, and mechanical properties, and the underlying mechanisms were revealed by the simulation. Scanning electron microscopy (SEM) combined with electron backscatter diffraction (EBSD) was used to investigate the influence of solidification parameters on the microstructure, and microhardness and tensile tests were performed to analyze the mechanical properties. Analyses combining FEM analysis and experiments will be carried out to investigate the relationship between processing parameters, and thermal history during solidification in laser additive manufacturing, and to investigate the correlation with the microstructure, resulting in mechanical properties.

## 2. Materials and Methods

### 2.1. L-PBF Process

**Figure 1:**
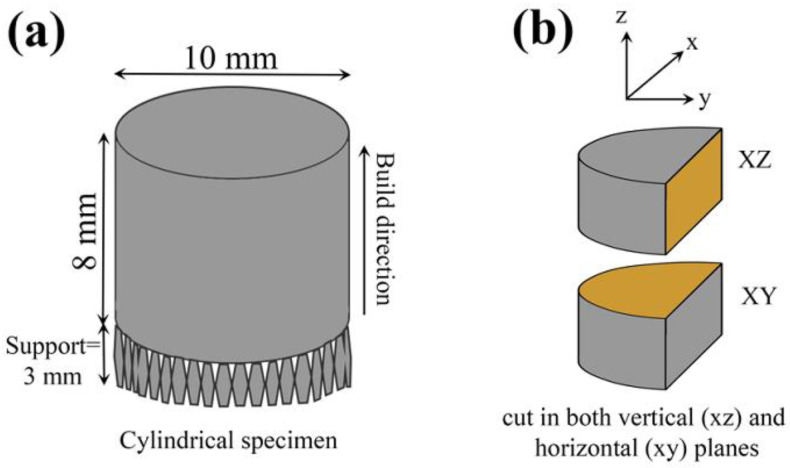
Schematic diagrams of (**a**) L-PBF HX cylindrical specimen and (**b**) horizontal (xy) and vertical (xz) regions for microstructural observation and hardness testing.

The gas atomized Hastelloy X (HX) powder (Amperprint^®^0228.074, Höganäs) with a mean diameter of 30 μm was used as the feedstock powder in this study. The chemical compositions of the Hastelloy X powder are listed in Table 1. All HX specimens were fabricated on a SUS304 substrate prepared with laser powder bed fusion (L-PBF) technology using the SLM280HL machine (Nikon SLM Solution, Germany) with a Gaussian laser beam with a maximum nominal power of 700 W and a focused beam diameter of 80 μm under an Ar atmosphere. The cylindrical specimens (diameter: 10 mm, height: 8 mm) were fabricated with a layer thickness of 30 μm, hatch spacing of 100 μm, and rotating scan vectors (90°) in each successive layer (Figure 1a), where the laser power and scanning speed are varied, as shown in Table 2. The combination of these process parameters is commonly evaluated by the energy density, which is given by the Equation (1).

(1) $$E=\frac{P}{v\times h\times l}$$

where E is the total energy density (J/mm^3^), P is the laser power (W), v is the scanning speed (mm/s), h is the hatch spacing (mm), and l is the layer thickness (mm).

### 2.2. Material Characterization Techniques

The microstructure of the L-PBF HX specimens was characterized using optical microscopy (OM, Keyance, USA), scanning electron microscopy (SEM: JSM-6010LA, JSM-7200F, JEOL, Japan), and electron backscatter diffraction (EBSD: JSM-7200F, JEOL, Japan). The cylindrical specimens prepared for OM, SEM, EBSD, and hardness testing were cut in both vertical (parallel to the building direction z: x–z plane) and horizontal (parallel to the x–y plane of laser scanning: x–y plane) sections by a wire cutting machine (Figure 1b). The cut specimens were mounted in resin using a hot mounting machine and then mechanically polished. The defect area rate (total area of pores, lack of fusion, and cracks) and mean grain size were measured by taking 25 images on the x–y and x–z planes of each specimen by OM and SEM operated at 20 kV, respectively. The Fiji ImageJ program was used to determine the defect area rate (%) and average grain size (μm) of each region. Grain orientation and texture were determined using the electron backscattered diffraction (EBSD) technique with an acceleration voltage of 15 kV and a step size of 0.2 μm.

### 2.3. Tests for Mechanical Properties

The microhardness distributions along the x–y and x–z axis directions of the specimens were measured using a Vickers hardness tester (AVK-A/AKASHI, Mitutoyo, Japan) at a load of 5 kg for 15 s. The test position was measured randomly at 10 points on each specimen to calculate the average.

**Figure 2:**
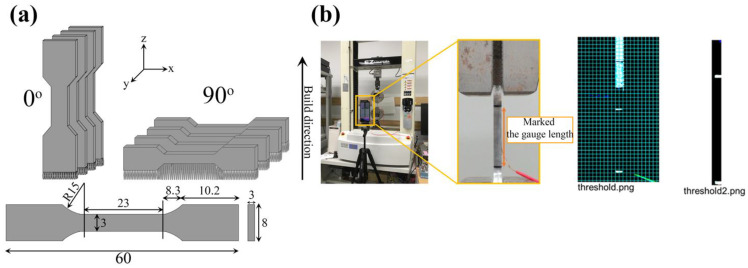
(**a**) Schematic diagram of L-PBF fabricated tensile specimens in different tensile directions and tensile specimen diameter (in mm) and (**b**) an image of the experimental method for strain estimation during tensile testing, using a smartphone video to record the deformation until failure and simulating the strain from the images.

The dog-bone tensile specimens were fabricated oriented along with the build direction (0°) and normal direction (90°) using a subset of the processing conditions used in the microstructural investigation. The tensile specimen process parameters and geometries are shown in Table 3 and Figure 2a, respectively. After printing, an annealing process was performed to remove residual stress from the tensile specimens. In the annealing process, the temperature was increased from room temperature to 303 K/s, held at 823 K for 1 h, and then cooled in a furnace. A wire electric discharge machine (AG360L, Sodick, Japan) was used to remove the specimens from the board. The surface of the shaped tensile specimen was polished with 180-600 SiC grit paper. Tensile tests were performed at room temperature using a compact universal table-top tensile machine (EZ graph, Shimadzu, Japan) with a strain gauge (KFEL-2-120-C1L3M2R, KFEM-1-120-C1L3M2R, Kyowa Electric Industry Co., Ltd., Japan). The maximum nominal of strain gauge on this study is 20%, but the elongation of HX specimens showed more than 20%, it can not measure the stress–strain curve to the end. Therefore, the strain was measured with a video (4K, 3840 × 2160 pixels) by smartphone. To determine the strain from the video, a line was drawn at the parallel length on the side of the specimen. The tensile specimen was attached to a tensile testing machine, a tripod was set up in front of the testing machine, and a video was recorded with a smartphone, as shown in Figure 2b. The video was recorded simultaneously with the start of the test started and stopped after the fracture occurred. Python (version 3.7.3) was used to determine the parallel line and line lengths from the images and to calculate the strain. Three tensile specimens were tested for each condition.

### 2.4. FEM Analysis

**Figure 3:**
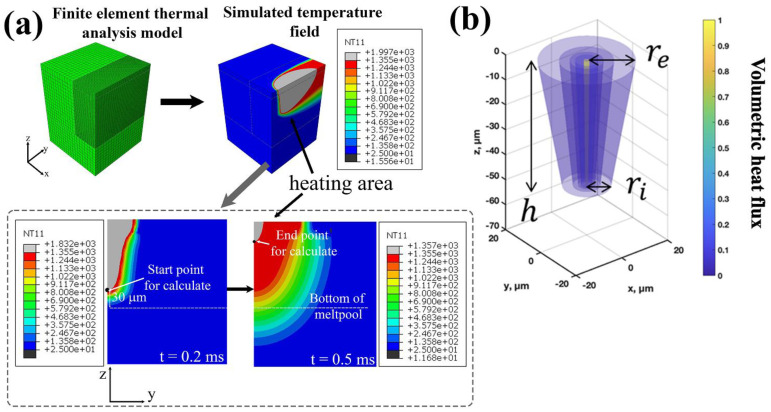
Finite element model employed for analysis (**a**) overall model and enlarged view of the heating area and temperature distribution with the calibrated shape parameters, and (**b**) heat flux distribution of the conical model.

To investigate the relationship between process conditions and structure from calculations, the cooling rate, temperature gradient, and solidification rate under different laser conditions were obtained by the finite element method (FEM) (Figure 3). According to the development of the heat source model by Kusano and Watanabe [13], the finite element model consists of a substrate and powder bed with a height of 2.03 mm and a width of 2.0 mm. The simulated powder layer thickness is 30 μm, which corresponds to the actual experimental powder layer. The initial temperature for this model was 25 °C, the top surface was a heat dissipated by radiation and convection (heating area), and the other surfaces were adiabatic boundaries, as shown in Figure 3a. The energy input region (heating area) was determined by the truncated cone heat source model, which depends on the laser power (P [W]) and scanning speed (v [m/s]), as shown in Figure 3b. The energy input by laser scanning was modeled as a moving volumetric heat flux (Q [J/m^2^s]). The heat flux distribution was presented as the truncated cone (represented as Equation (2) and shown in Figure 3b) [13,14]. The shape parameters $r_{e}$, $r_{i}$, and h were calibrated in the previous study [13], and depended on the laser power and scanning speed as described in Equations (3) and (4).

(2) $$Q=\frac{2AP}{\pi h(r_{e}^{2}+r_{e}r_{i}+r_{i}^{2})}exp(-2\frac{x^{2}+y^{2}}{r^{2}})$$

(3) $$r=r_{e}=r_{i}=38.2-44.5\frac{P}{v}$$

(4) $$h=-3.3+74.2\frac{P}{v}$$

The density was 4.11 kg/m^3^ for powder and 8.22 kg/m^3^ for bulk, which were constant, regardless of the temperature. The temperatures of the solidus and liquidus were set to T_S_ = 1533 K and T_L_ = 1628 K, respectively. The temperature distribution was obtained by the above analysis, and the cooling rate (GxR [K/s]), temperature gradient (G [K/m]), and solidification rate (R [m/s]) were calculated, according to Kusano and Watanabe [13,15].

## 3. Results

### 3.1. Optimization of L-PBF Parameters

**Figure 4:**
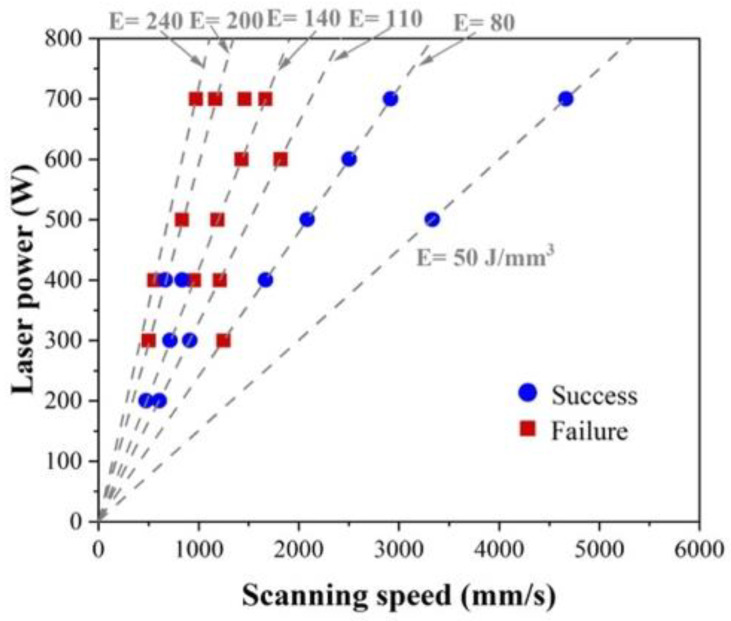
The effects of laser power and scanning speed on the success and failure of the HX samples during the deposition process.

Figure 4 shows the results of the optimal process parameter search, where the blue circle in the figure indicates points where the specimens could be completed to the end depending on the conditions, and the red squares indicate points where the samples failed due to surface soot, weak fusion, collapse, and balling defects during deposition. The results show that the combination of laser power and scan speed plays a key role in forming a uniform and continuous deposition and should provide sufficient energy to melt the powders. However, a scan speed that is too low and a laser power that is too high (high energy) create the defects during the deposition process, which lead to the incomplete fabrication of HX samples.

### 3.2. Effects of the Process Parameters on the Defects and Microstructure

**Figure 5:**
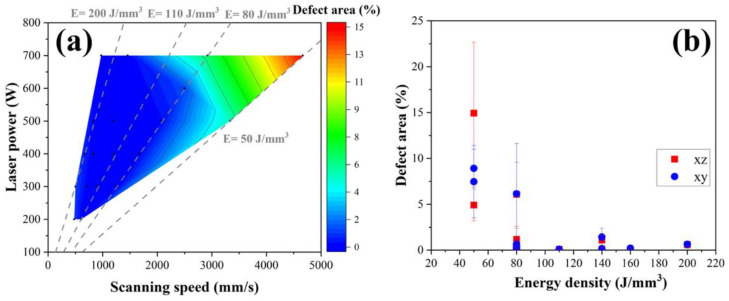
(**a**) Color map of the different laser power and scanning speed on the defect area of LPBF-fabricated HX, and (**b**) the relationship between defect area ratio and energy density in both vertical (xz) and horizontal (xy) directions.

**Figure 6:**
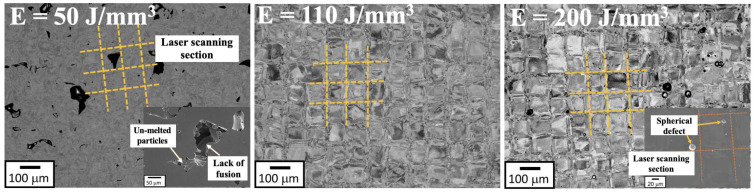
BSE images of xy cross-sections of the L-PBF HX manufactured with different energy densities.

The defect area rate (%), including the total area of pores, lack of fusion, and cracks was evaluated by comparing them according to laser power and scanning speed, as shown in Figure 5a. The value of E is determined by P and v, so both parameters are affected by the defect area rate (Figure 5b). The parameter conditions that ensure a low defect area rate below the 5% threshold are highlighted in the blue region of Figure 5a. The defect area rate of the LPBF-fabricated HX decreases with increasing laser power as the scanning speed value is decreased. It was observed that conditions with E below 100 J/mm^3^ resulted in more than 5% defect content along both the x–y and x–z axis directions of the specimens, indicating potential for low-quality specimen formation. Backscattered electron (BSE) micrographs of the as-polished XY cross-section of the HX specimens fabricated by different energy densities are shown in Figure 6. Lack of fusion defects and unmelted particles occur when insufficient energy (E = 50 J/mm^3^) is applied to the material. When the energy density is rose to 110 J/mm^3^, the lack of fusion defects disappears, but some spherical pores are observed at the high energy density of 200 J/mm^3^. The lack of fusion or large pores are caused by insufficient energy input, leading to the formation of the unfused phenomenon and unmelted area in the L-PBF HX specimen with low energy density, while the higher energy density caused more intense metal evaporation, resulting in the spherical pore in the L-PBF HX samples [16].

**Figure 7:**
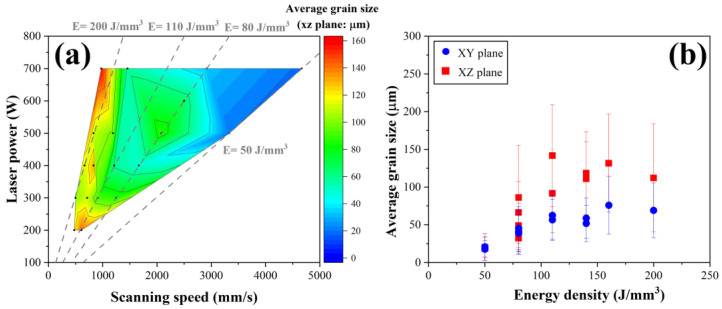
(**a**) Color map of different laser power and scanning speed on the average grain size of LPBF-fabricated HX; and (**b**) the relationship between the average grain size and energy density in both vertical (x–z) and horizontal (x–y) directions.

**Figure 8:**
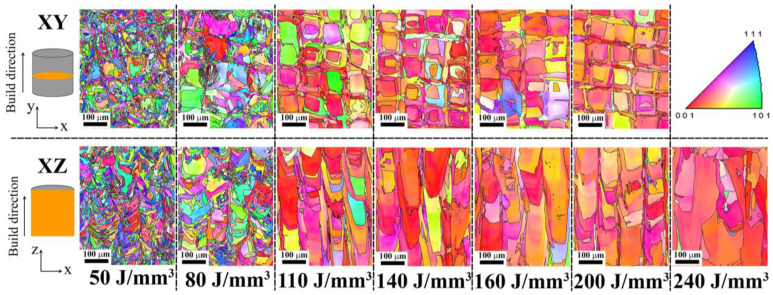
IPF maps showing the variation of crystallographic orientation projected in both vertical (xz) and horizontal (xy) directions along the build direction for different energy densities (J/mm^3^).

The EBSD scans were used to calculate microstructural measurements, including the average grain size and crystallographic texture, as shown in Figure 7 and Figure 8, respectively. The response contour plot for the influence of laser power and scanning speed on the relative average grain size is shown in Figure 7a, from which it can be directly seen that the average grain size of the specimens obviously increased at the low scanning speed with varying laser power, and then slightly decreased with the increasing scanning speed. Figure 7b shows the correlation between the energy density and the average grain size of the L-PBF HX alloy, which shows that the relative average grain size tends to increase, with the simultaneous increase in the energy density in the x–y and x–z planes. The measurements show that the HX samples have a more refined grain size (less than 50 μm) when the low energy density (<100 J/mm^3^) is applied. When the high energy density is applied, the average grain size becomes larger. EBSD crystallographic orientation maps of as-built HX exist at different energy densities along both the x–y and x–z cross-sections. In the x–y plane, which is perpendicular to the build direction, many checkerboard pattern grains appear, which is associated with the scanning strategy, and the x–z plane shows a microstructure composed of numerous columnar grains grown along the build direction, as shown in Figure 8. In terms of the crystallographic orientation, the random grain orientations with small grain size on x–y and x–z planes are detected in the L-PBF HX sample that applied the low energy density. On the other hand, the higher energy density leads to stronger directional cooling, which promotes strong texture along the <001> crystallographic direction of the larger grain parallel to both x–y and x–z planes similar to the reported by Hibino et al. [3]. In addition, the grain size and <001> single crystal of L-PBF HX also increases with the increasing energy density.

**Figure 9:**
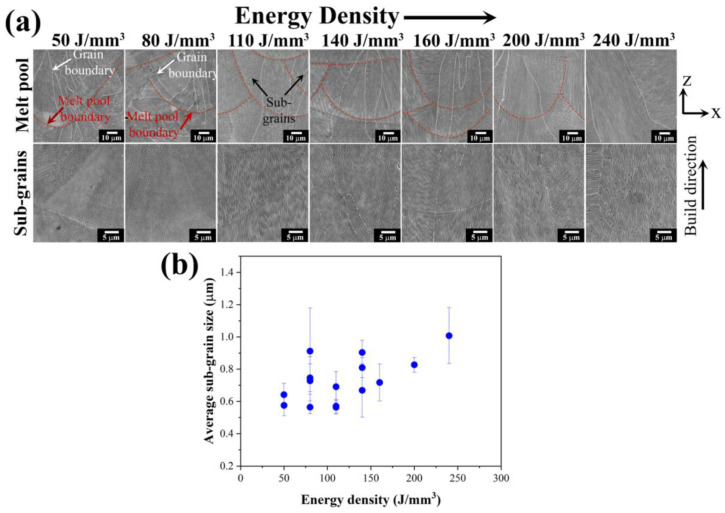
(**a**) SEM images showing the melt pool and sub-grain morphology on etched samples at different energy densities and (**b**) the measured average sub-grain width showing as a function of the energy density for L-PBF HX specimens.

The macro-grains in the melt pool had very complex substructures, and the SEM images of polished and chemically etched surfaces are shown in Figure 9a. The energy densities are varied, which contributes to the different melt pool sizes and sub-grain sizes. The melt pool geometry differs with deeper melting, which is attributed to rapid solidification due to increased energy input. In addition, the depth of the melt pool can affect the size of the grain structure. Melt pool solidification shows both the columnar type of grains and sub-grain structures at the micro level. At low energy densities (50–80 J/mm^3^), it can be observed that the specimen contains an equiaxed cellular and elongated morphology with a small width of cellular structure (less than 0.8 μm). When the energy densities are further increased to 100 J/mm^3^, it is shown that columnar growth is favored and epitaxial growth is dominant in the evolution of the microstructure throughout the build specimen, although not highly elongated. The width of the sub-grains increases slightly from 0.6 μm to 1 μm when the applied energy density is increased to 240 J/mm^3^, as shown in Figure 9b.

### 3.3. Effects of the Process Parameters on the Mechanical Properties

**Figure 10:**
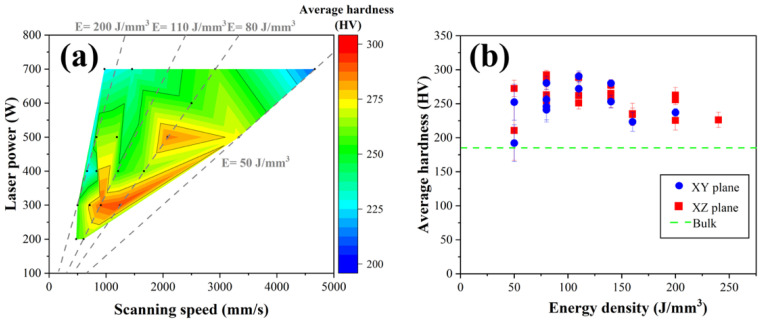
(**a**) Color map of different laser power and scanning speed on the average hardness value of LPBF-fabricated HX; and (**b**) the relationship between the average hardness value and energy density in both vertical (xz) and horizontal (xy) directions.

The overall effect of the process parameters on hardness from the collected data is shown in Figure 10. The microhardness measurement results vary between 200 and 300 HV among the specimens. Overall, these values are higher than those of the wrought HX alloy (180 HV [17]). In general, the hardness of a metal is inversely proportional to the grain size because the proportion of grain boundaries decreases. In addition, the defects cause the hardness to decrease. Therefore, the process parameters (laser power and scanning speed) for hardness vary, as shown in Figure 10a. However, the samples fabricated by low energy density was slightly highest hardness values (50–140 J/mm^3^) compared to the high energy density (>150 J/mm^3^), as shown in Figure 10b.

**Figure 11:**
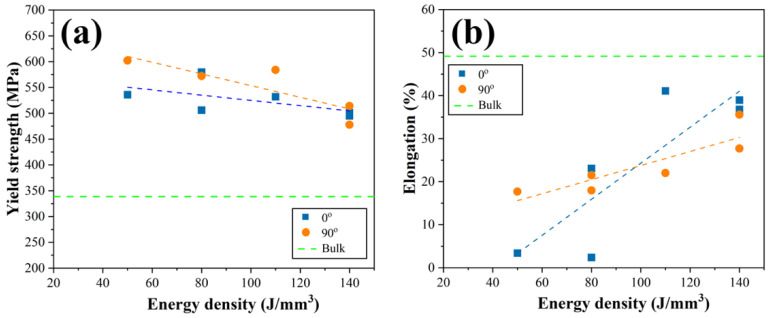
The relationship of the energy density to (**a**) yield strength and (**b**) elongation in both the 90° and 0° build directions.

In order to correlate the effect of the processing parameters on the tensile properties, the test was performed in different directions (0° and 90°). Figure 11 shows the variation of yield strength (YS) and elongation (EL) corresponding to the chosen energy density. The trend of the YS results on the specimens is similar in both the 0° and 90° directions, with the highest YS was observed at low energy density and decreasing with increasing the energy density (Figure 11a). The YS result for the 90° direction specimens is slightly higher than that for the 0° direction specimens due to the difference in the microstructure of the as-built specimen in the build direction. The elongation at break for each condition is shown in Figure 11b. Although the EL for all L-PBF HX samples is lower than the wrought HX comparison, there is an improvement in ductility when using high energy density in both build directions. Elongation is slightly higher for the 0° direction specimens tested at energy densities greater than 100 J/mm^3^ than for the 90° direction specimens at the same energy density.

### 3.4. FEM Simulation Results

**Figure 12:**
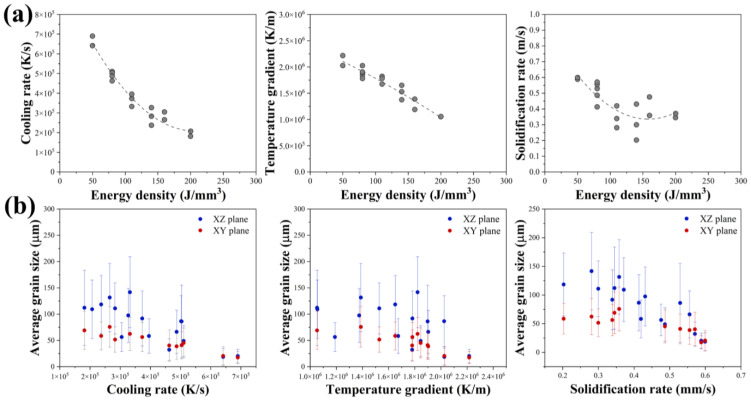
(**a**) Thermal history for a range of energy densities calculated from FEM analysis, (**b**) average grain size.

The solidified microstructure is determined by thermal conditions such as cooling rate (GxR), temperature gradient (G), and solidification rate (R) during solidification. The thermal analysis revealed that the cooling rate, temperature gradient, and solidification rate vary significantly at different energy densities within the melt pool (Figure 12a). It can be seen that three thermal conditions predict the same trend, which becomes smaller at a higher energy input. The relationship between thermal history and energy input is important to understand the control of the grain growth and morphology of the deposited materials. Figure 12b shows the average grain size as a function of the cooling rate, temperature gradient, and solidification rate for the HX alloy during the rapid solidification by the L-PBF process in both the x–y and x–z planes. It can be seen that the grain size is inversely proportional to the thermal history. In the non-equilibrium solidification, the thermal history is an important factor in determining the average grain size of the L-PBF specimens. When the cooling rate is low, a small increase in the cooling rate can result in a significant decrease in the grain size. When the cooling rate is high and continues to increase, the decrease in the grain size is not as pronounced.

## 4. Discussion

In this study, a combined experimental and numerical approach was used in order to evaluate the influence of the process parameters as a combination of laser power and scanning speed and the temperature evolution on the microstructure of the L-PBF HX alloy.

The temperature gradient, the cooling rate, and the solidification rate during the L-PBF process are important for controlling the grain size and the morphology in L-PBF specimens. The cooling rate varies in such a way due to the significant change in the solidification rate and the temperature gradient with the different energy input. The direction of grain growth is influenced by the temperature gradient, and the size of grain growth is controlled by the solidification rate, which, together with the cooling rate, controls the grain size of the solidified structure [6]. Heterogeneous nucleation during the rapid solidification of an alloy is also affected by constitutional supercooling. At high cooling rates (high GxR), the application of low energy density (<100 J/mm^3^) tends to increase the curvature fast cooling at the dendrite tips, resulting in an increased driving force for heterogeneous nucleation, which generates a highly textured with finer microstructure. The solidification microstructure evolves from unidirectional to equiaxed to dendritic with rapid cooling rate. The low cooling rate (low GxR) in high-energy-input specimens (>100 J/mm^3^) promotes a coarser structure and elongated shape due to the melt flow from the bottom to the top of the melt pool. The coarse grains are characteristic of a lower cooling rate due to the increasing heat accumulation from the high energy input, resulting in a remelting effect and grain growth in the layer-by-layer L-PBF process [18]. The growth direction of cells in the existence are well aligned with the local G in the previous layer, and the crystal will continue to grow epitaxially into the new layer without changing direction. It has favored a relatively stronger <001> crystal orientation along the build direction compared with the low energy input samples, which is consistent with the thermal gradient direction and also promotes the grain growth direction. These results are consistent with the study reported by Prasad et al. for HX [19] and Andreau et al. for 316 L [20] using the high energy density in the L-PBF process.

The mechanical properties including the strength, ductility, etc., of L-PBF specimens are closely related to the unique heterogeneous microstructure consisting of the solidification grains and the size of sub-grains developed during the rapid solidification of the L-PBF process. According to the Hall–Petch relationship, a refinement of the microstructural grain size is associated with a strengthening of the materials [21,22]. The dislocation of cellular grains can effectively strengthen the material by the dislocation movement and continuous flow of dislocation through the sub-grain boundaries. For the low energy density specimens, the hardness values were higher than those of the high energy density specimens due to the fine grain structure with the high dislocation density. Thus, it appears that the increase in strength caused by the increases in cooling rate and the decreases in sub-grain and grain size contribute to the strengthening. In addition, as the energy density increased, the YS tended to decrease but the EL increased, which can be explained by the grain morphology and grain size. The coarsening of the microstructure decreased as a result of the cooling rates due to the heat flux having the lowest number of grain boundaries, thus resulting in fewer barriers to the dislocation movement, which reduced the strength of the material. In general, the Young’s modulus varies with the interatomic distances (E[111] > E[110] > E[100]) in an FCC system [23,24,25]. The YS was found to decrease and the EL to increase in specimens with a strong <100> texture. The L-PBF HX specimens with coarser grains including columnar grains have lower strength but higher plasticity (EL), while those with finer grains have higher strength and lower plasticity. The tensile property was changed by several factors including microstructure, crystallographic texture and defects, the same as Hibino et al. [3], who reported that the yield stress, ultimate tensile stress, and elongation can be selected according to the mechanical requirements by changing the crystallographic orientation and grain boundary density through the L-PBF process. In addition, the build with different orientation of the tensile specimens was reflected in the tensile properties. The grain growth typically takes place in a directional manner, usually transverse to the building direction, resulting in microstructure and texture influence; therefore, we started with the angle between the columnar oriented grains, which were through the building direction and tensile axis [26,27]. The 90° specimen built along the building direction had slightly higher YS and EL, which showed a good combination of strength and plasticity, similar to the results of Xiong et al. [28] and Tomus et al. [10]. Mechanical properties performed over a wide range of grain sizes indicate that this thermal history, including temperature gradient and cooling rate effects by varying process parameters, which is primarily related to increases in the lattice resistance to flow and is strongly associated with grain boundary strengthening [26,27,29]. The anisotropy of the mechanical properties of the specimens with different building directions was not determined by the columnar texture, but by the distribution of the melt pool boundary on the load bearing surface [28].

## 5. Conclusions

In this study, the L-PBF processing window established for Hastelloy X was determined by experiments and compared with numerical FEM modeling. The relationship between the L-PBF processing parameters, defects, microstructure, and mechanical properties was investigated. The following conclusions were reached:

1. Insufficient energy input resulted in porous structures with the a of fusion defects, while excessive energy input led to some spherical pores by metal evaporation.
2. When a high energy input is used, the cooling rate during L-PBF is reduced, thereby increasing the cell and grain size. The lowest thermal gradient promotes the epitaxial growth and columnar grain, resulting in the formation of both a morphological and crystallographic texture along the building direction.
3. The finer grain with a high dislocation density caused by the high cooling rate in the low-energy-input L-PBF process was responsible for the high hardness values together with the tensile strength. The heterogeneous structure with fine grains in various L-PBF processing parameters of the HX alloy is responsible for the higher mechanical properties when compared to that produced by the traditional process.

Highly desirable microstructural features of the HX alloy could be achieved by controlling the processing parameters in the L-PBF process, which would result in various combinations of strength, ductility, and toughness for L-PBF parts. The microstructure property–process relationship provides a method for selecting suitable mechanical properties for L-PBF HX alloy under different service environments.

## Figures and Tables

**Table 1:** Chemical composition of Hastelloy X (wt%) by EDS.

Ni	Fe	Cr	Co	Mo	W	C
Bal.	18.1	22.1	1.6	9.0	0.7	0.06

**Table 2:** Parameters of L-PBF process.

Parameters	
Laser type	Gaussian beam
Laser power (W)	200–700
Scanning speed (mm/s)	476–4667
Hatch spacing (μm)	100
Layer thickness (μm)	30
Laser diameter (μm)	70
Scanning strategy	90° rotation

**Table 3:** Parameters of L-PBF process for build the tensile specimens.

Conditions	Energy Density (J/mm^3^)	Laser Power (W)	Scanning Speed (mm/s)	Hatch Spacing (μm)	Layer Thickness (μm)	Laser Type
**0°, 90°** **3 samples/** **condition**	50	500	3333	100	30	Gaussian beam
	80	700	2917			
	80	500	2083			
	110	300	909			
	140	200	476			
	140	300	714			

## Data Availability

The original contributions presented in the study are included in the article, and further inquiries can be directed to the corresponding author.
